# METTL14-dependent maturation of pri-miR-17 regulates mitochondrial homeostasis and induces chemoresistance in colorectal cancer

**DOI:** 10.1038/s41419-023-05670-x

**Published:** 2023-02-21

**Authors:** Kangyue Sun, Lu Chen, Yiwen Li, Bing Huang, Qun Yan, Changjie Wu, Qiuhua Lai, Yuxin Fang, Jianqun Cai, Yongfeng Liu, Junsheng Chen, Xinke Wang, Yuxuan Zhu, Shuyu Dong, Jieyu Tan, Aimin Li, Side Liu, Yue Zhang

**Affiliations:** 1 Guangdong Provincial Key Laboratory of Gastroenterology, Department of Gastroenterology, Nanfang Hospital, Southern Medical University, Guangzhou, China; 2 Department of Gastroenterology, Zhuhai People’s Hospital (Zhuhai Hospital Affiliated with Jinan University), Zhuhai, China

**Keywords:** Colorectal cancer, Cancer therapeutic resistance

## Abstract

miR-17-5p has been found to be involved in the proliferation and metastasis of colorectal cancer (CRC), and *N*^6^-methyladenosine (m^6^A) modification is the most common RNA modification in eukaryotes. However, whether miR-17-5p contributes to chemotherapy sensitivity in CRC via m^6^A modification is unclear. In this study, we found that overexpression of miR-17-5p led to less apoptosis and lower drug sensitivity in vitro and in vivo under the 5-fluorouracil (5-FU) treatment, which indicated miR-17-5p led to 5-FU chemotherapy resistance. Bioinformatic analysis suggested that miR-17-5p-mediated chemoresistance was associated with mitochondrial homeostasis. miR-17-5p directly bound to the 3’ untranslated region of Mitofusin 2 (*MFN2*), leading to decreased mitochondrial fusion and enhanced mitochondrial fission and mitophagy. Meanwhile, methyltransferase-like protein 14 (METTL14) was downregulated in CRC, resulting in lower m^6^A level. Moreover, the low level of METTL14 promoted the expression of pri-miR-17 and miR-17-5p. Further experiments suggested that m^6^A mRNA methylation initiated by METTL14 inhibits pri-miR-17 mRNA decay via reducing the recognition of YTHDC2 to the “GGACC” binding site. The METTL14/miR-17-5p/MFN2 signaling axis may play a critical role in 5-FU chemoresistance in CRC.

## Introduction

Colorectal cancer (CRC) is a major public health issue worldwide. There were estimated to be over 1.8 million new CRC cases and nearly 0.9 million CRC-related deaths worldwide in 2020, ranking this cancer third in incidence and second in mortality among all cancers [1]. Although immunotherapy is becoming a potential treatment, due to the low incidence of mismatch repair deficiency in CRC and poor clinical response to checkpoint immunotherapy, chemotherapy remains the typical treatment for advanced CRC [2, 3].

Fluorouracil (5-FU), which inhibits the enzymatic activity of thymidylate synthase during DNA replication [4], is an essential component of systemic chemotherapy for CRC in the palliative and adjuvant settings [5]. However, almost half of the patients diagnosed with metastatic CRC are resistant to 5-FU-based chemotherapy, and the five-year survival rate is only slightly greater than 12% [5]. Therefore, it is essential to develop a better understanding of the mechanism of 5-FU chemotherapy resistance in CRC, and the development of effective molecular approaches to enhance the chemotherapy response rate remains an urgent clinical need.

MicroRNAs (miRNAs), which are small noncoding RNAs that regulate the translation of various genes, play important roles in carcinogenesis and cancer development, diagnosis and prognosis. The miR-17-92 cluster is a classic miRNA family that has been reported to participate in cancer chemoresistance, for example, RNA-binding protein heterogeneous nuclear ribonucleoprotein A2/B1 (HNRNPA2B1) may affect the prognosis of esophageal cancer by regulating the miR-17-92 cluster [6]. miR-17-5p, which belongs to the oncogenic miR-17-92 cluster, has been reported to be associated with chemoresistance in renal cancer, nonsmall cell lung cancer and cervical cancer [7–9]. Previously, we reported that overexpression of miR-17-5p promoted CRC metastasis and established an oncogenic microenvironment [10]. However, the way in which miR-17-5p affects chemoresistance in CRC remains unclear, and the details of the mechanism need to be elucidated.

The *N*^6^-methyladenosine (m^6^A) modification is the most common RNA modification in eukaryotes. It regulates the cleavage, transport, localization, stability and translation of RNA at the posttranscriptional level [11–14], all of which can be modulated by “writers”, “erasers”, and “readers” [15]. Methyltransferase-like protein 14 (METTL14), a member of the methylation “writers” complex (METTL14, METTL3 and WTAP), has been shown to regulate the proliferation and metastasis of CRC [16–18]. Previous study has shown that METTL3 can increase the sensitivity of gastric cancer to mTOR inhibitors by promoting the maturation of the miR-17-92 cluster [19]. However, there is little research on the regulation of METTL14 in cancer chemotherapy resistance. Indeed, m^6^A mRNA methylation is recognized by “readers”, like YTH domain-containing proteins (YTHDFs and YTHDCs), which preferentially bind an RR(m^6^A)CU (R = G or A) consensus motif. YTHDC2 is one of the “reader” proteins. The role of YTHDC2 in mRNA biology is controversial: on the one hand, YTHDC2 can inhibit mRNA expression at the transcriptional level by degrading mRNAs [20, 21]; on the other hand, YTHDC2 can also promote mRNA translation via the elongation-promoting effect of CDS methylation [22]. Although the functions of METTL14 and YTHs in various organisms have been partly clarified, the mechanisms through which m^6^A regulates CRC chemoresistance require further exploration.

Mitochondrial homeostasis plays a critical role in maintaining the physiological functions of cells and is involved in diverse biological processes across every type of cancer [23–27], including CRC [28–30]. Key nodes in mitochondrial homeostasis involve multiple processes, including mitochondrial fusion and fission [31] and mitophagy [32]. Recently, a study reported that mitochondrial mitophagy induced by fission resulting in fragmentation led to hepatocellular carcinoma cell survival in the context of TAE/TACE treatment [24]. To date, many studies have focused on the relationship between mitochondrial dynamics and chemotherapy resistance; however, there is little research on the regulation of CRC, particularly with respect to mitochondrial homeostasis. Therefore, it is necessary to study the effect and mechanism of mitochondrial homeostasis related to chemotherapy resistance induction in CRC, which may become a breakthrough in this field.

In the present study, we found that miR-17-5p promoted 5-FU resistance in CRC in vitro and in vivo; specifically, miR-17-5p enhanced mitochondrial fission and mitophagy, thereby regulating mitochondrial function and leading to 5-FU resistance. miR-17-5p upregulation is attributable to m^6^A modification mediated by METTL14 and YTHDC2, which leads to reduced degradation of pri-miR-17. Taken together, our results provide a detailed mechanism connecting m^6^A modification of a pri-miRNA, mitochondrial homeostasis and chemotherapy resistance, which may serve as attractive therapeutic targets in CRC.

## Materials and methods

### Patients and tissue specimens

A total of 43 tumor tissues and adjacent normal colon tissues were obtained from CRC patients at Nanfang Hospital of Southern Medical University (Guangzhou, P.R. China). The procedure for human specimen collection was approved by the Ethics Committee of Southern Medical University. Patient consent was obtained before the start of the study.

### Cell lines

Human CRC cell lines (HCT116, LoVo, SW480, SW620, Caco2, HT29, and RKO) and normal colonic epithelial cells (FHC, NCM460) were purchased from the Cell Bank of Type Culture Collection (CBTCC, China Academy of Sciences, Shanghai, China). Cell lines were authenticated by short tandem repeats profiling and confirmed to be mycoplasma negative. HCT116 cells were cultured in RPMI 1640 medium (Gibco, USA), and other cells were cultured in Dulbecco’s modified Eagle medium (DMEM) (Gibco, USA) supplemented with 10% fetal bovine serum (FBS; ExCell Bio, China) at 37 °C in a 5% CO_2_ atmosphere.

### Transient transfection and lentiviral infection

Specific miR-17-5p mimic, miR-17-5p inhibitor, and METTL14-specific siRNA construct were synthesized by GenePharma (China). For plasmid construction, METTL14 was purchased from Vigenebio (China). Cells were transfected with the indicated siRNA or plasmid using Lipofectamine 3000 reagent (Invitrogen, USA) following the manufacturer’s instructions. The sequences for the constructs listed above are shown in Supplementary Table S1.

To construct cell lines overexpressing exogenous miR-17-5p, full-length hsa-miR-17-5p (GeneChem, China) and the empty control were cloned into the lentiviral vector system (puro-miR-17-5p and puro-NC). The lentiviruses were transfected into SW480 cells according to the manufacturer’s instructions. Puro-miR-17-5p and puro-NC cells were screened with puromycin (2 μg/ml; BioSharp, China). For cell lines that overexpress or knock down METTL14, METTL14 OE (Obio, China) and sh*METTL14* (Obio) were cloned into the lentiviral vector system (neo-METTL14 OE, neo-NC; neo-sh*METTL14*, neo-shNC). The sequence of sh*METTL14* is same as si-*METTL14* shown in Supplementary Table S1. After transfected the lentiviruses, these cells were screened with G418 (800 μg/ml; BioSharp). To obtain miR-17-5p/METTL14 co-expressing cells, neo-METTL14 lentiviruses were transfected to miR-17-5p-overexpressing CRC cell lines and also screened with G418 (800 μg/ml; BioSharp).

### Western blot (WB) and immunohistochemistry (IHC) analysis

WB analysis was performed using anti-METTL14, anti-MFN2, anti-FIS1, anti-DRP1, anti-BAX, anti-BCL2, and anti-Caspase7/cleaved-Caspase7 (26158-1-AP, 12186-1-AP, 10956-1-AP, 12957-1-AP, 50599-2-Ig, 12789-1-AP, 27155-1-AP; 1:1000; Proteintech, USA); anti-LC3A/B and anti-p62 (306019, 380612; 1:1000; ZenBio, China); anti-PARP and anti-cleaved-PARP (9542, 5625; 1:1000; CST, USA); and anti-YTHDC2 (ab220160, 1:1000, Abcam, UK) antibodies. The loading control was a mouse anti-GAPDH monoclonal antibody (60004-1-Ig; 1:10000; Proteintech).

For IHC analysis, consecutive tumor sections with a thickness of 4 μm were prepared. Then, deparaffinization and antigen retrieval were performed following the manufacturer’s instructions. These sections were incubated with anti-Ki-67, anti-MFN2 (27309-1-AP; 12186-1-AP; Proteintech) and anti-Caspase7 (T40049S; Abmart, China) antibodies. After incubated with secondary antibodies (PV-6001, ZSGB-BIO, China), the sections were visualized with a DAB chromogenic agent (ZLI-9017, ZSGB-BIO) and observed under a microscope.

### RNA m^6^A quantification

Total RNA was extracted from cells using TRIzol reagent. Total RNA m^6^A quantification was performed using an m^6^A RNA Methylation Assay Kit (ab185912; Abcam) according to the manufacturer’s instructions. Total RNA binds to microbars by RNA binding solution. RNA with m^6^A is captured by m^6^A antibodies and detected by detection antibodies. After the detection signal is enhanced, the absorbance at 450 nm wavelength is read by a microplate spectrophotometer for colorimetric quantification. The amount of m^6^A is proportional to the measured OD value.

### RNA immunoprecipitation (RIP) and methylated RNA immunoprecipitation (MeRIP)

A RIP assay was performed with the RNA Immunoprecipitation (RIP) Kit (BersinBio, China) in accordance with the manufacturer’s protocol. In brief, cells were lysed in RIP lysis buffer, and then the whole-cell extract was incubated with anti-DGCR8 (Proteintech) antibody, followed by magnetic beads. Finally, the coprecipitated RNAs were extracted and subjected to qRT–PCR using primers for pri-miR-17 and normalized to the input.

For m^6^A RNA-binding assays, the Methylated RNA Immunoprecipitation (MeRIP) Kit (BersinBio) was used. In brief, RNA was extracted with TRIzol reagent and fragmented by ultrasonication. The fragmented RNA was incubated with an anti-m^6^A antibody (91261, Active Motif, USA) followed by magnetic beads. The enrichment of m^6^A-containing mRNA was then analyzed through qRT–PCR and normalized to the input.

### RNA stability

Cells were seeded in 12-well plates at 2 × 10^5^ cells per well, incubated overnight and treated with actinomycin D (ActD; 5 μg/ml, Selleck). The cells were harvested after treatment for the indicated times (0, 15, 30, 60, and 120 min), and RNA was isolated from these cells for RT–PCR analysis.

### RNA isolation, reverse transcription, and quantitative real-time PCR (RT–PCR)

See in Supplementary Materials and Methods.

### Cell apoptosis assay

See in Supplementary Materials and Methods.

### Cell growth

See in Supplementary Materials and Methods.

### EdU proliferation assay

See in Supplementary Materials and Methods.

### Animals and a tumor growth assay

See in Supplementary Materials and Methods.

### Analysis of public databases

See in Supplementary Materials and Methods.

### Determination of the mitochondrial DNA (mtDNA) copy number

See in Supplementary Materials and Methods.

### Mitochondrial membrane potential and an immunofluorescence assay

See in Supplementary Materials and Methods.

### Mitochondrial morphology

See in Supplementary Materials and Methods.

### Statistical analysis

Data were analyzed using GraphPad Prism 8 (GraphPad Software, USA). Correlations between two genes were determined using Spearman correlation analysis. Other data were computed using two-tailed student’s t-test or one-way analysis of variance (ANOVA). Data are presented as the mean, and *P* < 0.05 was considered statistically significant.

## Results

### miR-17-5p reduces 5-FU-stimulated CRC apoptosis in vitro and in vivo

**Fig. 1:**
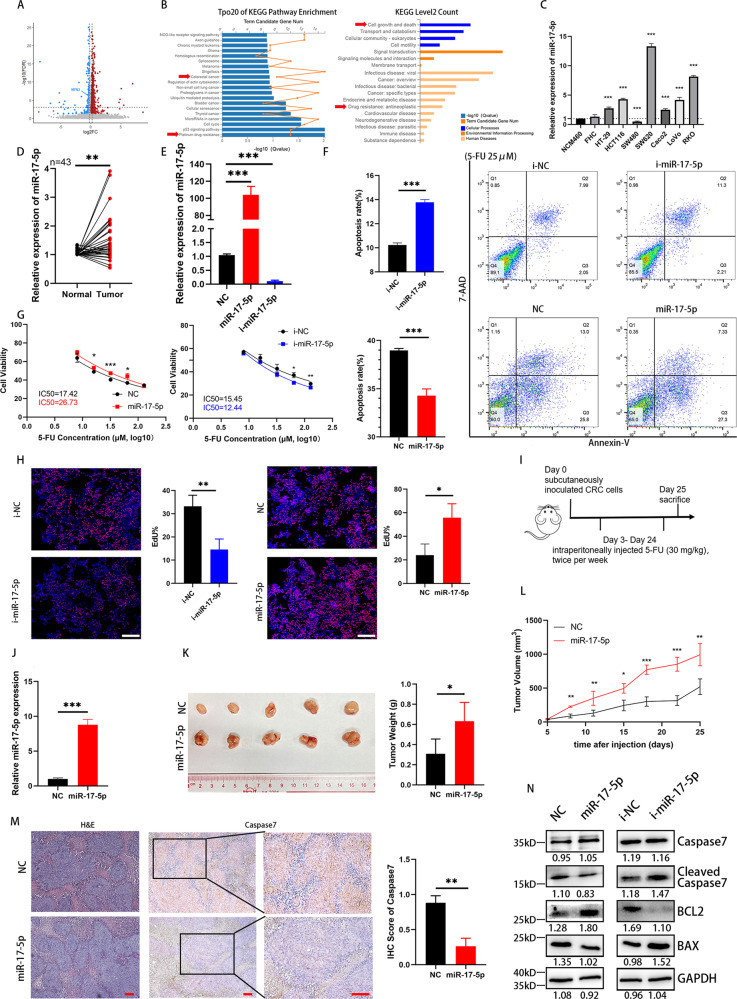
miR-17-5p reduces the 5-FU-induced apoptosis. **A** Up- and down-regulated genes in HCT116 cells after miR-17-5p overexpression. **B** Top 20 KEGG pathways enrichment and KEGG secondary classification of miR-17-5p-overexpressed HCT116 cells. **C** Relative expression of miR-17-5p in normal intestinal epithelial cells and CRC cell lines. **D** Relative expression of miR-17-5p in 43 pairs of CRC tissues from a clinical cohort. *P* = 0.0019. **E** Overexpression and knockdown efficiency of miR-17-5p in HCT116 cells. Both *P* < 0.0001. **F** Annexin V-PE/7AAD apoptosis assay of HCT116 cells with different miR-17-5p expression levels after 5-FU treatment. P (i-NC vs. i-miR-17-5p) < 0.0001; P (NC vs. miR-17-5p) = 0.0004. **G** The 5-FU IC50 of HCT116 cells with different miR-17-5p expression levels. **H** EdU proliferation assay of HCT116 cells with different miR-17-5p expression levels after 5-FU treatment. P (i-NC vs. i-miR-17-5p) = 0.0032; P (NC vs. miR-17-5p) = 0.0106. **I** Procedure for treatment of xenografted nude mouse models. **J** Overexpression efficiency of stably transfected miR-17-5p CRC cells. *P* < 0.0001. **K** The tumor sizes of xenograft after 6 times 5-FU treatment and the weights in different groups. *P* = 0.0301. **L** The changes of tumors volume during the treatment of 5-FU. **M** H&E and immunohistochemistry analysis of Caspase7 in two groups. *P* = 0.0021. **N** Western blot analysis of Caspase7, cleaved Caspase7, BCL2 and BAX protein expression levels in different groups of HCT116 cells treated with 5-FU. Results shown were representative of at least 3 independent experiments. Statistical significance in (**D**), (**F-H**), (**J-M**) was assessed by student’s t-test. Statistical significance in (**C**), (**E**) was determined by one-way ANOVA with Dunnett’s multiple comparisons test. *, *P* < 0.05; **, *P* < 0.01; ***, *P* < 0.001. Error bars, SD. Scale bars, 400 μm in (**H**), 100 μm in (**M**). The EdU ratio, IHC score and grey value of protein bands have been quantified by Image J.

In a previous study, we reported that miR-17-5p plays an oncogenic role in promoting CRC proliferation and metastasis, but how it influences the chemotherapy sensitivity is still elusive. We performed RNA sequencing (RNA-seq) of HCT116 cells with overexpression of miR-17-5p or a scrambled negative control. As a result, 385 genes were found to be upregulated, while 169 genes were negatively correlated with miR-17-5p overexpression (Fig. 1A). The top 20 enriched pathways are listed in Fig. 1B, among which cell growth and death and antineoplastic drug resistance stood out as significantly enriched pathways, suggesting a vital role for miR-17-5p in chemotherapy sensitivity. We detected miR-17-5p expression in different colorectal cell lines; compared with cell lines with relatively low metastatic potential, such as SW480 and HCT116, the SW620 cell line, which has high metastatic potential, shown significant overexpression of miR-17-5p (Fig. 1C). RT–PCR analysis verified that miR-17-5p was overexpressed in 43 human CRC tissues compared with adjacent normal mucosal tissues (Fig. 1D). To evaluate the biological function of miR-17-5p in CRC cell lines, we used miR-17-5p mimics (miR-17-5p) and an inhibitor (i-miR-17-5p) to carry out an in vitro analysis with HCT116 cells. RT–PCR was utilized to confirm the transfection efficiency in HCT116 cells (Fig. 1E). To further determine whether miR-17-5p influences apoptosis in CRC, cell apoptosis was studied using flow cytometry. As shown in Fig. 1F, in the context of induction with 5-FU, miR-17-5p overexpression reduced the apoptosis rate of HCT116 cells. The CCK-8 method was used to determine the IC50 values of 5-FU for the parental line HCT116 and cell lines transfected with the mimics or inhibitor. Cell growth curves were established to calculate the population doubling time. Overexpression of miR-17-5p significantly upregulated the IC50 value of 5-FU from 17.42 μM to 26.73 μM (Fig. 1G). An EdU proliferation assay performed with HCT116 cells after 5-FU treatment shown that miR-17-5p overexpression promoted tumor proliferation, while knockdown inhibited it (Fig. 1H).

In vivo, nude mice with xenografts were treated with 5-FU (Fig. 1I). As shown in Fig. 1J-L, the tumors in the overexpression xenograft group grew faster and were larger than those in the control group. Furthermore, IHC shown that the overexpression group exhibited a lower positive Caspase7 staining tumor cell percentage than the NC group, which indicated that there was less apoptosis in the overexpression group (Fig. 1M). Western blot results shown that the overexpression of miR-17-5p upregulated BCL-2 and downregulated cleaved-Caspase7 and BAX protein expression levels compared with control expression (Fig. 1N).

These results shown that miR-17-5p enhanced CRC tumor growth both in vivo and in vitro, indicating that miR-17-5p decreased 5-FU-stimulated CRC apoptosis.

### miR-17-5p modulates mitochondrial dynamics by targeting MFN2 expression

**Fig. 2:**
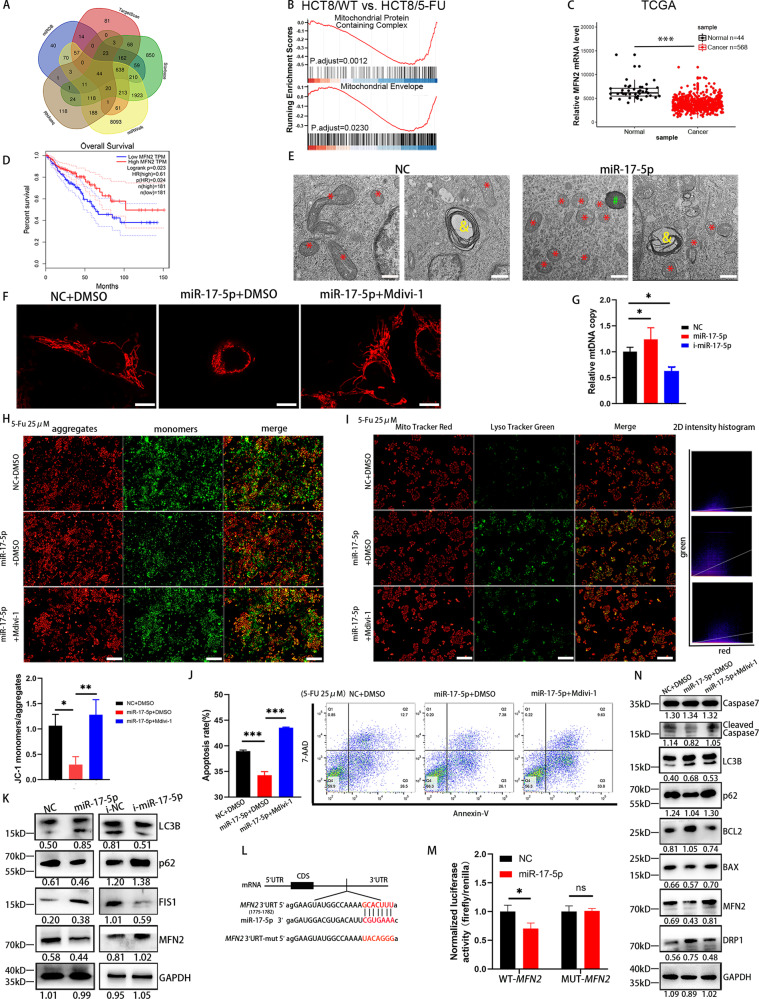
miR-17-5p reduces the 5-FU-induced apoptosis via mitophagy dynamics and mitophagy. **A** The target genes of miR-17-5p were predicted by online databases such as TargetScan, miRDB, miRWalk and StarBase as well as RNA-seq. **B** Gene set enrichment analysis (GSEA) of GSE81005. HCT8/WT, HCT-8 human CRC wild type cells; HCT8/5-FU, its 5-FU-induced resistant cell line. **C** Expression of MFN2 in the TCGA CRC cohort. **D** Analysis of overall survival of MFN2 from GEPIA databases. **E** Transmission electron microscopy (TEM) images of mitochondria in control cells and miR-17-5p overexpression cells. Red *, mitochondria; green #, lysosomes; yellow &, autolysosome. **F** Mitochondrial morphology of control HCT116 cells and miR-17-5p overexpression cells with or without Mdivi-1 used under a confocal microscopy. **G** Relative mtDNA copy of HCT116 cells with different miR-17-5p expression levels. P (NC vs. i-miR-17-5p) = 0.0106; P (NC vs. miR-17-5p) = 0.0424. **H** Mitochondrial membrane potential was indicated by JC-1 staining. Red fluorescence: JC-1 aggregates, Green fluorescence: JC-1 monomers. P (NC + DMSO vs. miR-17-5p + DMSO) = 0.0120; P (miR-17-5p + DMSO vs. miR-17-5p + Mdivi-1) = 0.0037. **I** Colocalization (yellow puncta) of lysosomes (Lyso Tracker Green) and mitochondria (Mito Tracker Red). The yellow puncta indicated mitochondria-containing autolysosomes. Manders coefficients for green-to-red colocalization were 0.287, 0.593, 0.340 respectively. **J** Annexin V-PE/7AAD apoptosis assay of control and miR-17-5p overexpression HCT116 cells with or without Mdivi-1 used. Both *P* < 0.0001. **K** Western blot analysis of LC3B, p62, FIS1 and MFN2 protein expression levels in different miR-17-5p expression levels HCT116 cells. **L** Prediction of miR-17-5p binding site in the 3′UTR region of MFN2 based on TargetScan. **M** miR-17-5p and a luciferase vector encoding the wild-type or mutant MFN2 3′UTR region were cotransfected into HCT116 cells, the relative luciferase activity was measured. P (WT-*MFN2*: NC vs. miR-17-5p) = 0.0253. **N** Western blot analysis of Caspase7, cleaved Caspase7, LC3B, p62, BCL2, BAX, MFN2 and DRP1. Results shown were representative of at least 3 independent experiments. Statistical significance in (**M**) was assessed by student’s t-test. Statistical significance in (**G**-**J**) was determined by one-way ANOVA with Dunnett’s multiple comparisons test. *, *P*<0.05; **, *P*<0.01; ***, *P*<0.001. Error bars, SD. Scale bars, 500 nm in (**E**), 10 μm in (**F**), 400μm in (**H**) and **I**. The EdU ratio, IHC score and grey value of protein bands have been quantified by Image J, and the grey value of LC3B shown the ratio of LC3II/LC3I.

To gain insight into the mechanism by which miR-17-5p regulates chemotherapy resistance in CRC, we searched for potential targets of miR-17-5p using online databases such as TargetScan, miRDB, miRWalk and StarBase and intersected the targets with the RNA-seq results. Of all the genes identified, 44 were predicted to be target genes of miR-17-5p (Fig. 2A). Gene set enrichment analysis (GSEA) was performed to evaluate the biological relevance of the identified target genes. The results suggested that “mitochondrial protein containing complex” and “mitochondrial envelope” are important to the biological function of 5-FU resistance (Fig. 2B). Interestingly, Mitofusin 2 (*MFN2*) was identified as a target gene (Fig. 1A) and has been reported to play a pivotal role in mitochondrial dynamics. We used the TCGA to assess the *MFN2* mRNA level, and *MFN2* expression was significantly lower in CRC tissues than in normal epithelial tissues (Fig. 2C). Human clinical data from the Gene Expression Profiling Interactive Analysis (GEPIA) server based on TCGA data revealed that lower *MFN2* levels were associated with a decreased survival rate (Fig. 2D).

To further identify the relationship between miR-17-5p and MFN2, we conducted additional experiments. Transmission electron microscopy (TEM) was applied to observe the structural changes in the mitochondria and autophagosome. As shown in Fig. 2E, the miR-17-5p overexpression group shown a distinctly fragmented mitochondrial morphology compared with the control group, which indicated that miR-17-5p increased fission and decreased mitochondrial fusion. Mdivi-1, an inhibitor of mitochondrial fission, whose target is Dynamin-Related Protein 1 (DRP1) GTPase, was used to do the rescue experiments. Similar to the results in Fig. 2E, Mito Tracker staining indicated that the shape of the mitochondria in the miR-17-5p overexpression group was fragmented and appeared as granular dots, which could be recovered to a fused state after the application of Mdivi-1, with a tubular shape, suggesting that miR-17-5p induces mitochondrial division and suppresses mitochondrial fusion (Fig. 2F). The mtDNA copy number, a biomarker of mitochondrial function, was evaluated by RT–PCR. The results shown that miR-17-5p increased mitochondrial fission and that i-miR-17-5p produced the opposite effect **(**Fig. 2G). The monomer of JC-1 fluoresces green, whereas the polymer fluoresces red; and an increase in green fluorescence indicates a decrease in the mitochondrial membrane potential, which is a sign of premature cell death. When we used 5-FU to stimulate the miR-17-5p overexpression and control groups, overexpression of miR-17-5p was shown to inhibit the formation of JC-1 monomers; however, Mdivi-1 attenuated this inhibitory effect. These results suggested that miR-17-5p reduced apoptosis in CRC cells induced by 5-FU in a mitochondrial fission-dependent manner (Fig. 2H). As shown in Fig. 2I, increases in mitochondria-containing autolysosomes (yellow puncta) were observed when miR-17-5p overexpression, but Mdivi-1 weakened the mitophagy-enhancing effect. The apoptotic status was also assessed by flow cytometry; compared with control treatment, miR-17-5p significantly inhibited apoptosis in HCT116 cells, while Mdivi-1 reversed the antiapoptotic effect (Fig. 2J).

After transient transfection of HCT116 cells with miR-17-5p or i-miR-17-5p, WB analysis was employed to evaluate MFN2 expression (Fig. 2K). Furthermore, the levels of the LC3-II/LC3-I were markedly increased and those of the protein p62 were significantly decreased in the miR-17-5p group (Fig. 2K), indicating accelerated mitophagosome formation (an event in mitophagy). Furthermore, mitochondrial fission protein (FIS1) was also elevated in the miR-17-5p group. According to the miR-17-5p binding site in *MFN2* identified by TargetScan (Fig. 2L), we established *MFN2*-WT and *MFN2*-MUT luciferase reporter plasmids. A dual-luciferase reporter assay shown that in the presence of miR-17-5p, the luciferase activity of *MFN2*-WT was decreased, whereas that of *MFN2*-MUT was not changed (Fig. 2M). The result suggests that miR-17-5p directly targets the 3’UTR region of MFN2 and down-regulates its expression. Additionally, miR-17-5p significantly downregulated the expression of the proapoptotic proteins BAX, cleaved Caspase7, and p62 and upregulated the expression of the antiapoptotic protein BCL2 as well as that of the autophagy biomarker LC3B compared to control treatment. Combining Mdivi-1 with miR-17-5p reduced the expression of DRP1, enhanced the expression of MFN2, and shown less pronounced effects on apoptosis compared with miR-17-5p alone (Fig. 2N). Furthermore, compared with overexpression of miR-17-5p alone, co-expression of MFN2 resulted in increased apoptosis of CRC cells, decreased IC50 values, and increased expression of BAX, Caspase7, cleaved Caspase7, PARP and cleaved PARP (Sup Fig. 1).

These results indicate that miR-17-5p-dependent chemotherapy resistance in CRC is mediated by controlling mitochondrial homeostasis.

### Knockdown of METTL14 reduces 5-FU-stimulated CRC apoptosis

m^6^A modification has been reported in a number of studies on cancers, and METTL14 is one of the most important regulatory proteins. According to the TCGA database and GSE41657, METTL14 was expressed at low levels in adenoma and further decreased in colorectal carcinoma, exhibiting synchrony with CRC progression. Similarly, RT–PCR analysis confirmed that METTL14 was expressed at lower levels in 43 human CRC tissues compared with adjacent normal mucosal tissues. In addition, GEPIA shown that lower METTL14 expression was related to shorter overall survival and disease-free survival (Sup Fig. 2). Above results indicate that METTL14 is downregulated in CRC, and associated with poor clinical outcomes.

**Fig. 3:**
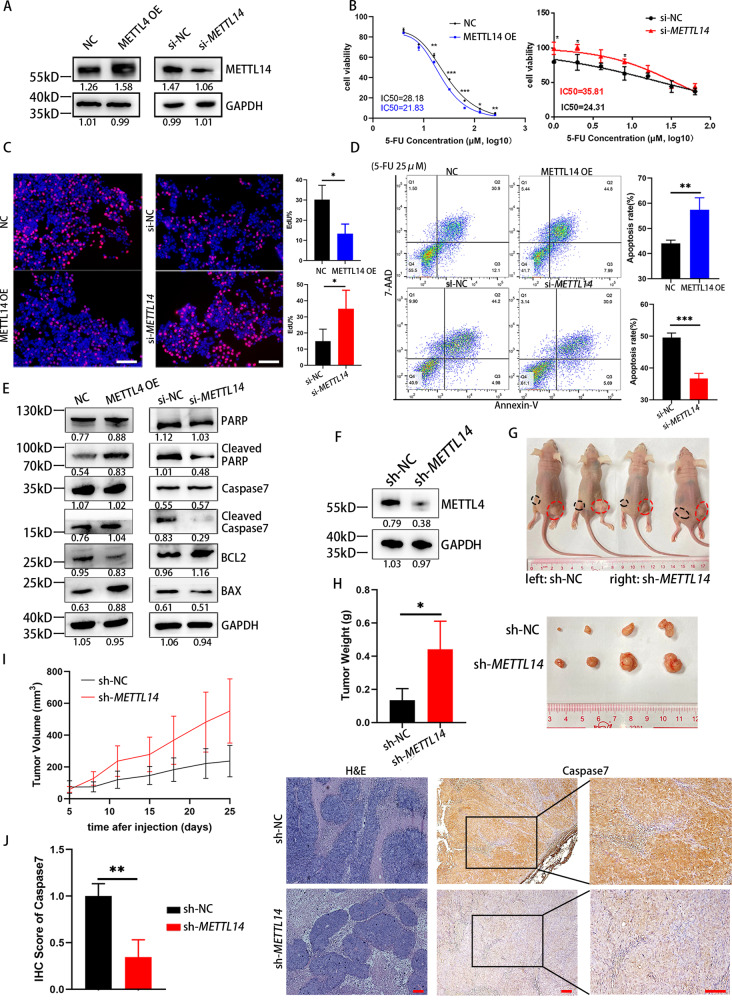
METTL14 promotes the 5-FU-induced apoptosis. **A** Overexpression and knockdown efficiency of METTL14 in HCT116 cells. **B** The 5-FU IC50 of HCT116 cells in different groups. **C** EdU proliferation assay of HCT116 cells with different METTL14 expression levels after 5-FU treatment. P (NC vs. METTL14 OE) = 0.0265; P (si-NC vs. si-*METTL14*) = 0.0379. **D** Annexin V-PE/7AAD apoptosis assay of HCT116 cells with different METTL14 expression levels after 5-FU treatment. P (NC vs. METTL14 OE) = 0.0095; P (si-NC vs. si-*METTL14*) = 0.0005. **E** Western blot analysis of PARP, Cleaved PARP, Caspase7, Cleaved Caspase7, BCL2 and BAX protein expression levels in different METTL14 expression levels HCT116 cells treated with 5-FU. **F** Knockdown efficiency of stably transfected METTL14 CRC cells. **G** Nude mice were implanted with tumor cells subcutaneously. **H** Tumor sizes and weights of xenograft after 6 times 5-FU treatment. *P* = 0.0360. **I** The changes of tumors volume during the treatment of 5-FU. **J** H&E and immunohistochemistry analysis of Caspase7 levels in two groups. *P* = 0.0077. Results shown were representative of at least 3 independent experiments. Statistical significance in (**B-D**), (**H**), (**J**) was assessed by student’s t-test. *, *P*<0.05; **, *P*<0.01; ***, *P*<0.001. Error bars, SD. Scale bars, 400 μm in (**C**), 100 μm in (**J**). The IHC score and grey value of protein bands has been quantified by Image J.

To study the influence of METTL14 expression levels on CRC cells, we successfully knocked down METTL14 expression in HCT116 cells using specific siRNAs or overexpressed METTL14 using a METTL14-carrying plasmid (Fig. 3A). As shown in Fig. 3B, METTL14 overexpression significantly attenuated the IC50 value of 5-FU for HCT116 cells (28.18 μM vs. 21.83 μM), whereas knockdown of METTL14 increased the IC50 value (24.31 μM vs. 35.81 μM). Next, we validated the effects of METTL14 overexpression or knockdown on the proliferation of HCT116 cells using EdU staining. Confocal microscopy demonstrated that METTL14 overexpression significantly decreased the proliferation of HCT116 cells (Fig. 3C). Furthermore, METTL14 enhanced the apoptosis rate of HCT116 cells observed with 5-FU induction, while knockdown of METTL14 significantly suppressed the apoptosis rate (Fig. 3D). To further validate these findings, we performed WB analysis to assess biomarkers of the apoptotic process (Fig. 3E). The analysis shown that cleaved PARP, cleaved Caspase7 and BAX were upregulated in the METTL14 overexpression group, and BCL2 was downregulated; the METTL14 knockdown group shown the opposite results. The above results suggest that METTL14 overexpression promotes apoptosis of CRC cells, while METTL14 knockdown is the opposite. We also knocked down METTL14 in SW480 cells through stable transfection with a *METTL14*-knockdown lentivirus. WB analysis was performed to validate the knockdown efficiency (Fig. 3F). Then, we assessed the physiological relevance of METTL14 to CRC colonization and chemotherapy sensitivity in vivo, and stable cells with modified METTL14 expression were injected subcutaneously. As shown in Fig. 3G, SW480 cells with METTL14 deficiency exhibited marked CRC tumor growth, as shown by the tumor weight and growth curves of these groups compared with those of the control groups (Fig. 3H and I). Moreover, IHC results shown that METTL14 knockdown resulted in appreciably decreased Caspase7 staining in tumor cells of the xenografts (Fig. 3J), which indicated that there was less apoptosis in the METTL14 knockdown group. Collectively, these results indicate the critical role of METTL14 in modulating chemosensitivity to 5-FU in CRC.

### METTL14 suppresses the expression of miR-17-5p via m^6^A modification

**Fig. 4:**
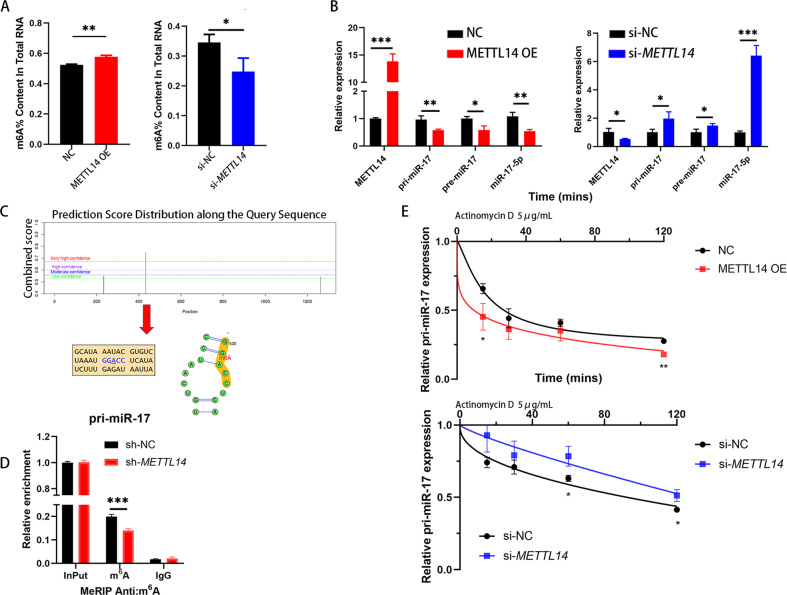
METTL14 regulates miR-17-5p expression through m^6^A modification. **A** Colorimetric quantification of m^6^A levels in HCT116 cells after METTL14 knockdown (*P* = 0.0322) or overexpression (*P* = 0.0012). **B** Relative expression of pri-miR-17, pre-miR-17 and miR-17-5p after METTL14 knockdown (*P* = 0.0335, 0.0313, 0.0322, 0.0002 respectively) or overexpression (P = 0.0001, 0.0084, 0.0121, 0.0049 respectively). **C** Possible m^6^A modification sites on the pri-miR-17 predicted by SRAMP database. **D** MeRIP and MeRIP-qPCR analysis demonstrating pri-miR-17 m^6^A modification in sh-NC and sh-*METTL14* cells, P = 0.0009. **E** qPCR analysis of pri-miR-17 levels in different HCT116 cells groups after actinomycin D treatment. P (NC vs. METTL14 OE, 15 mins = 0.0272, 120 mins = 0.0021); P (si-NC vs. si-*METTL14*, 60 mins = 0.0209, 120 mins = 0.0175). Results shown were representative of at least 3 independent experiments. Statistical significance in (**A**), (**B**), (**D**), (**E**) was assessed by student’s t-test. *, *P* < 0.05; **, *P* < 0.01; ***, *P* < 0.001. Error bars, SD.

Mounting evidence indicates that m^6^A plays crucial roles in miRNA genesis and maturation. As shown in Fig. 4A, HCT116 cells with METTL14 knockdown exhibited reduced m^6^A levels, whereas HCT116 cells overexpressing METTL14 exhibited higher m^6^A levels than control cells. Furthermore, METTL14 inhibited the expression of miR-17-5p, pre-miR-17, and pri-miR-17, while knockdown of METTL14 reversed these effects (Fig. 4B). RIP results shown that DGCR8 was bound to pri-miR-17 in HCT116 cells (Sup Fig. 3).

We next searched for consensus motifs and identified the RRACH sequence in pri-miR-17 as the m^6^A modification site, which was supported by bioinformatic analysis results (Fig. 4C). In addition, the MeRIP experiment shown that the m^6^A modification existed on pri-miR-17 mRNA was decreased when METTL14 knockdown (Fig. 4D). To verify how METTL14 regulated pri-miR-17 expression, we performed RNA stability experiments via actinomycin D. As shown in Fig. 4E, we found that ectopic expression of METTL14 reduced the expression of pri-miR-17, which was transcriptionally blocked by the transcription inhibitor actinomycin D, while silencing METTL14 upregulated the expression of pri-miR-17. Together, these data indicate that there are sites allowing m^6^A modification mediated by METTL14 on pri-miR-17 mRNA that have the potential to regulate the expression of pri-miR-17 in CRC cells.

### METTL14 knockdown enhances pri-miR-17 mRNA stability via an m^6^A-YTHDC2-dependent pathway

**Fig. 5:**
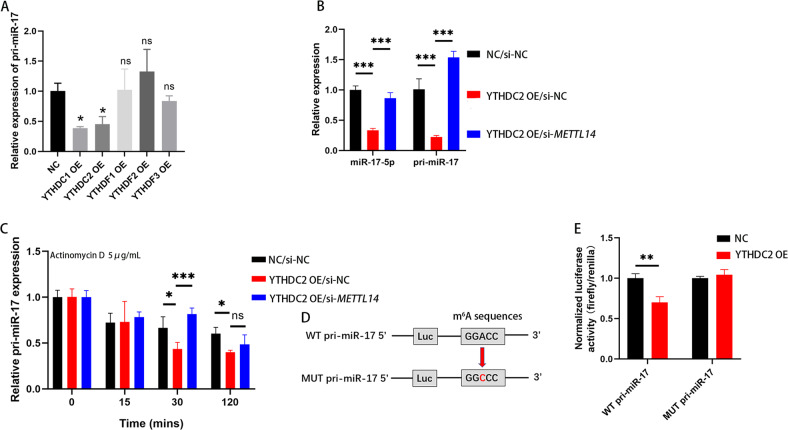
METTL14 knockdown enhances pri-miR-17 mRNA stability via an m^6^A-YTHDC2-dependent pathway. **A** Relative expression of pri-miR-17 after HCT116 cells transfected YTHDC1-2, YTHDF1-3 plasmids. P (NC vs. YTHDC1 OE) = 0.0198; P (NC vs. YTHDC2 OE) = 0.0385. **B** Relative expression of pri-miR-17 and miR-17-5p after HCT116 cells transfected YTHDC2 plasmids and cotransfected si-*METTL14* and YTHDC2 plasmids. **C** PCR analysis of pri-miR-17 levels in different HCT116 cells groups after actinomycin D treatment. **D** Luciferase vector encoding the wild-type or m^6^A consensus sequence mutated pri-miR-17 were constructed. **E** YTHDC2 induced the posttranscriptional repression of pri-miR-17 in HCT116 cells. P (WT pri-miR-17: NC vs. YTHDC2 OE) = 0.0046. Results shown were representative of at least 3 independent experiments. Statistical significance in (**E**) was assessed by student’s t-test. Statistical significance in (**A–C**) was determined by one-way ANOVA with Dunnett’s multiple comparisons test. *, *P* < 0.05; **, *P* < 0.01; ***, *P* < 0.001. Error bars, SD. The grey value of protein bands has been quantified by Image J.

m^6^A modification plays roles in pri-miRNA maturation, decay, stability and translocation to the cytoplasm. m^6^A can be recognized by the YT521-B homology (YTH) domain-containing proteins, which subsequently regulates RNA signaling pathways. Five YTH domain-containing proteins, YTHDC1–2 and YTHDF1–3, were overexpressed in HCT116 cells **(**Sup Fig. 4B**)**. The level of mRNA pri-miR-17 was only related to YTHDC1 and YTHDC2 (Fig. 5A). Between them, YTHDC2 has been reported to promote mRNA degradation in both oncological [20] and non-neoplastic diseases [21]. PCR analysis were performed to confirm the cotreatment with YTHDC2 OE and NC or si-*METTL14* regulated the expression of pri-miR-17 and miR-17-5p. When overexpressing YTHDC2, pri-miR-17 and miR-17-5p were downregulated, while knocking down METTL14 diminished these decreases (Fig. 5B). Next, we explored whether YTHDC2 affects the stability of pri-miR-17 with m^6^A modification. Data shown that pri-miR-17 degraded rapidly under actinomycin D induction when YTHDC2 overexpression alone, but was rescued via cotransfection of si-*METTL14* and YTHDC2 in HCT116 cells (Fig. 5C). Furthermore, we generated a mutant sequence of the m^6^A binding site in pri-miR-17 that could not bind the m^6^A modification for use in subsequent luciferase reporter assays (Fig. 5D). As demonstrated in Fig. 5E, the YTHDC2 plasmid significantly decreased the luciferase activity in HCT116 cells transfected with the wild-type pri-miR-17 sequence, whereas the luciferase activity in HCT116 cells transfected with the pri-miR-17 mutant was not obviously altered. These findings indicate that YTHDC2 can interact with pri-miR-17 transcripts and promotes its decay by recognition and binding m^6^A mRNA modification. Interestingly, we found that HCT116 cells with METTL14 knockdown shown decreased protein levels of YTHDC2. In contrast, HCT116 cells overexpressing METTL14 shown increased expression of YTHDC2, however YTHDC2 cannot regulate levels of METTL14 in CRC cells (Sup Fig. 4). Further researches on the regulation between YTHDC2 and METTL14 are needed.

In conclusion, these results suggest that the m^6^A “writer” METTL14 increases the level of m^6^A modification of pri-miR-17, which is then recognized by an m^6^A “reader”, the YTHDC2. However, whether other molecules are involved in this process remains to be further explored.

### METTL14 depletion significantly enhances 5-FU sensitivity via the miR-17-5p/MFN2 axis

**Fig. 6:**
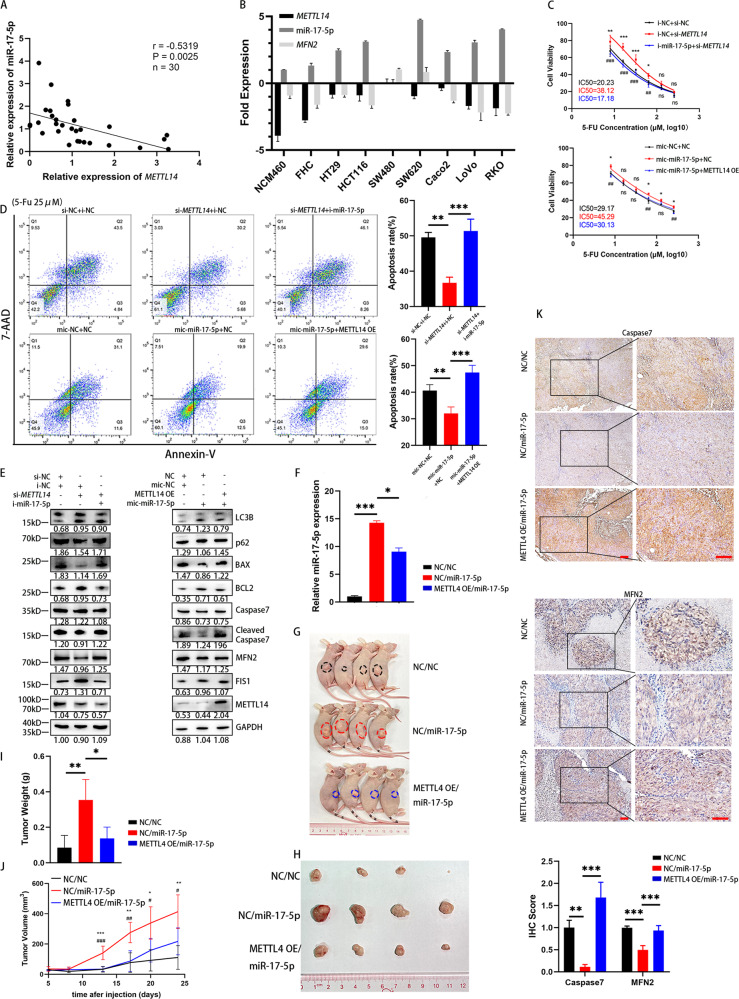
METTL14 depletion significantly enhances 5-FU resistance via the miR-17-5p/MFN2 axis. **A** Correlation between METTL14 and miR-17-5p expression levels in CRC tissues with linear regression lines (*n* = 30). **B** RT-PCR analysis of METTL14, miR-17-5p and MFN2 expression levels in normal intestinal epithelial cells and CRC cell lines. **C, D** Rescue experiments on METTL14 and miR-17-5p: IC50 and Annexin V-PE/7AAD apoptosis of 5-FU in HCT116 cells. In (**D**), P (si-*METTL14* + i-NC vs. si-NC + i-NC) = 0.0010; P (si-*METTL14* + i-NC vs. si-*METTL14* + i-miR-17-5p) = 0.0005. P (mic-NC + NC vs. mic-miR-17-5p + NC) = 0.0096; P (mic-miR-17-5p +NC vs. mic-miR-17-5p + METTL14 OE) = 0.0005. **E** Western blot analysis of LC3B, p62, BAX, BCL2, Caspase7, cleaved Caspase7, MFN2, FIS1, METTL14 of rescue experiments. **F** Transfection efficiency of cell lines with stable co-expression of miR-17-5p and a control vector (NC/miR-17-5p, *P* = 0.0007) or METTL14 (METTL14 OE/miR-17-5p, P = 0.0190). **G** Nude mice were implanted with tumor cells subcutaneously. **H**, **I** Tumor sizes and weights of xenograft in three groups after 6 times 5-FU treatment. In (**I**), P (NC/NC vs. NC/miR-17-5p) = 0.0030, P (NC/miR-17-5p vs. METTL14 OE/miR-17-5p) = 0.0111. **J** The changes of tumors volume during the treatment of 5-FU. **K** Immunohistochemistry analysis of Caspase7 and MFN2 in three groups. P Caspase7 (NC/NC vs. NC/miR-17-5p) = 0.0049; P Caspase7 (NC/miR-17-5p vs. miR-17-5p/METTL14 OE) = 0.0003; P MFN2 (NC/NC vs. NC/miR-17-5p) ˂0.0001; P MFN2 (NC/miR-17-5p vs. miR-17-5p/METTL14 OE) = 0.0002. Results shown were representative of at least 3 independent experiments. Statistical significance in (**A**) was assessed by Pearson correlation analysis. Statistical significance in (**C**), (**D**), (**F**), (**I**), (**J**-**K**) was determined by one-way ANOVA with Dunnett’s multiple comparisons test. *, *P*<0.05; **, *P*<0.01; ***, *P*<0.001. Error bars, SD. Scale bars, 100 μm in (**K**). The IHC score and grey value of protein bands have been quantified by Image J, and the grey value of LC3B shown the ratio of LC3II/LC3I.

To verify the influence of METTL14 on miR-17-5p expression in vivo, we tested the expression of METTL14 and miR-17-5p in 30 CRC tissues using RT–PCR. Spearman rank correlation analysis revealed a negative correlation between the METTL14 and miR-17-5p levels (Fig. 6A). Then, we evaluated the expression of METTL14, miR-17-5p, and MFN2 in several CRC cell lines (Fig. 6B). In contrast to METTL14 knockdown, which enhanced IC50 values and suppressed the apoptosis rate, miR-17-5p knockdown significantly rescued the IC50 values and apoptosis rates of HCT116 cells under 5-FU stimulation. Correspondingly, overexpression of miR-17-5p suppressed apoptosis levels, while ectopic expression of METTL14 repressed this suppression (Fig. 6C, D). Moreover, WB assays revealed that downregulation/upregulation of METTL14 led to expression changes in the mitochondrial dynamic proteins FIS1 and MFN2, autophagy-associated proteins LC3B, p62, and the apoptosis-associated proteins BAX, BCL2, Caspase7 and cleaved Caspase7, while co-transfection of i-miR-17-5p/mic-miR-17-5p counteracted the effects of METTL14 (Fig. 6E). Furthermore, we next explored whether METTL14 modulates CRC 5-FU sensitivity via the METTL14/miR-17-5p/MFN2 axis. We generated a SW480 cell line with stable co-expression of miR-17-5p and a control vector or METTL14 OE, and RT–PCR was used to test the transfection efficiency (Fig. 6F). We then used these cells to generate a mouse xenograft model of 5-FU sensitivity (Fig. 6G). As shown in Fig. 6H and I, the miR-17-5p group exhibited larger tumors, while METTL14 opposed the enhancing effects on tumor mass and weight under the treatment of 5-FU. Approximately 2 weeks after subcutaneous implantation of these cells into mice, slower tumor growth was observed in the METTL14 OE/miR-17-5p groups than in the NC/miR-17-5p group (Fig. 6J). Additionally, IHC analysis of the Caspase7 and MFN2 expression levels in tumor cells of xenografts revealed that the miR-17-5p group exhibited lower levels of Caspase7 and MFN2 than the negative control group, which indicated that there was less apoptosis in the miR-17-5p group, and MFN2 was downregulated by miR-17-5p; however, these effects were abolished by overexpressing METTL14 (Fig. 6K). Collectively, the results reveal that the METTL14/miR-17-5p/MFN2 axis may play an oncogenic role in 5-FU sensitivity in CRC.

## Discussion

CRC is a highly aggressive and malignant cancer, and although substantial improvements have been made in the therapeutic interventions for CRC, surgery and chemotherapy are the most basic treatments. However, chemotherapy resistance remains a critical problem in CRC therapy, as this resistance produces poor survival rates and low quality of life in CRC patients; thus, it is essential to clarify the chemoresistance mechanisms and enhance the chemosensitivity of CRC. There is increasing evidence that noncoding RNAs play important roles in regulating chemotherapy sensitivity in CRC [33–36], which involves protective autophagy [34] and inhibition of crucial cell cycle steps [36]. In our previous paper, we shown that miR-17-5p, a member of the miR-17-92 cluster, is related to the premetastatic microenvironment of CRC [10]. This cluster is located on chromosome 13q31.3 in the third intron of the C13orf25 gene and contains 6 miRNAs (miR-17, miR-18, miR-19a, miR-20a, miR-19b-1 and miR-92-1). Recent studies have reported that miR-17-5p, compared to all the miRNAs in the miR-17-92 cluster, is overexpressed in different chemo-resistant cancers, including renal cancer [7], non-small cell lung cancer [8] and cervical cancer [9]. In this paper, we demonstrated that higher miR-17-5p levels induced stronger proliferation in CRC cells, inhibited cancer cell apoptosis, and increased the IC50 of 5-FU. In vivo, subcutaneous tumors overexpressing miR-17-5p grew faster and exhibited less apoptosis under 5-FU induction, suggesting that miR-17-5p reduced the chemosensitivity to 5-FU in CRC. The results of immunohistochemical staining for Ki-67 are shown in Sup Fig. 5.

Next, combining the GSEA and RNA-seq results, as well as the miRNA target gene database, mitochondrial quality control (MQC) and mitochondrial fusion genes were considered to be involved in the mechanism. As an important means for preserving mitochondrial homeostasis, MQC mechanisms are a series of adaptive responses that preserve mitochondrial structure and function; they include mitochondrial fission, mitochondrial fusion, mitophagy and mitochondria-dependent cell death. A growing body of research has elucidated the relationship between MQC and tumor progression in many types of cancers. Importantly, the efficacy of tumor chemotherapy is influenced by mitochondrial fission/fusion and mitophagy. Chemotherapeutic drugs impair cellular homeostasis, including damaging subcellular structures such as the mitochondria, to lead to apoptosis. Cancer cells can rapidly clear damaged mitochondria through mitophagy, thereby protecting tumors from chemotherapy [37, 38]. An earlier study shown that hepatitis C virus (HCV) perturbs mitochondrial dynamics by promoting mitochondrial fission and mitophagy, which attenuates HCV-induced apoptosis [39]. In hepatocellular carcinoma, it was observed that mitophagy induced by mitochondrial fission protects tumor cells from apoptosis under TAE/TACE treatment [24]. In our study, similar mechanisms for cell survival by increasing mitochondrial fission and promoting mitophagy were discovered. miR-17-5p down-regulated the expression of one MQC-related specific gene, *MFN2*, impairing mitochondrial fusion. Meanwhile, FIS1-mediated mitochondrial fission and mitophagy increased, which ultimately led to 5-FU chemoresistance. Significantly, we observed that the above phenomena were reversed when the mitochondrial fission inhibitor Mdivi-1 was administered, indicating a mechanism by which miR-17-5p modulates the mitochondria via MFN2.

m^6^A is a ubiquitous form of RNA modification in both coding RNAs and noncoding RNAs, which has been reported to be involved in the proliferation, metastasis and chemoresistance of CRC. Most of the studies shown that m^6^A “writer” METTL14 functions as a proliferation and metastasis suppressor in CRC [16–18]. However, another research implicated that METTL14-mediated m^6^A modification contributes to the up-regulation of pleckstrin homology-like domain, family B, member 2 (PHLDB2), which mediates resistance in latent metastasis CRC [40]. Up to now, there has been no literature on the involvement of METTL14 in CRC chemoresistance through regulation of miRNA. Our findings provide insights into whether and how METTL14 regulates miR-17-5p via m^6^A modification to promote 5-FU resistance in CRC.

m^6^A modification of primary miRNAs is generally thought to be associated with promoting their maturation [41, 42]. However, we found that after knocking down METTL14, both the overall levels of m^6^A in CRC and the binding of m^6^A on pri-miR-17 were decreased, while the expression levels of pri-miR-17 and miR-17-5p increased. Based on these results, we hypothesized that m^6^A modification via METTL14 may participate in maintaining pri-miR-17 stability instead of promoting maturation or translocation. Indeed, RNA stability experiments confirmed that pri-miR-17 was more susceptible to degradation when METTL14 was overexpressed, and vice versa.

However, the m^6^A reader is the protein that ultimately determines the posttranscriptional effects of m^6^A modifications on RNA. Previous studies have confirmed that YTH domain-containing proteins, such as YTHDF3 [43], YTHDF2 [18], and YTHDF1 [44], impact the occurrence and development of CRC. YTHDF1 [45] and YTHDF3 [46, 47] always promote the post-transcriptional regulation of its targets. YTHDC2, YT521-B homology domain containing 2, is believed to be the final member of the YTH protein family, functioning as a reader to suppress liver steatosis and lung adenocarcinoma [20, 21]. Mechanistically, YTHDC2 bounds to m^6^A-modified mRNAs and thereafter decrease their mRNA stability. We also noticed that YTHDC2 downregulated the expression of pri-miR-17. Thus, the effect of YTHDC2 to pri-miR-17 was further investigated.

Our data indicated that after overexpressing YTHDC2, both pri-miR-17 and miR-17-5p were downregulated in CRC cells, while knocking down METTL14 diminished these decreases. RNA degradation experiments confirmed that pri-miR-17 degraded rapidly upon YTHDC2 overexpression but was rescued via METTL14 knockdown. Therefore, affecting the stability of miR-17-5p’s primary transcripts (pri-miR-17) is one of the mechanisms by which m^6^A regulates its level. However, it cannot be ruled out that there may be other forms of regulation, thus further investigation is needed for more details. Moreover, the dual-luciferase reporter assay system suggested that YTHDC2 binds directly to the A site on the GGACC motif of pri-miR-17. Although our results support the functions of METTL14 and YTHDC2 in regulating pri-miR-17 mRNA expression, the detailed mechanisms of m^6^A modification remain to be elucidated in the future.

In clinical tissue samples, the expression levels of miR-17-5p were negatively correlated with METTL14. In vivo and in vitro rescue assays demonstrated that the METTL14/miR-17-5p/MFN2 signaling axis affected the sensitivity of CRC to 5-FU chemotherapy.

## Conclusions

**Fig. 7:**
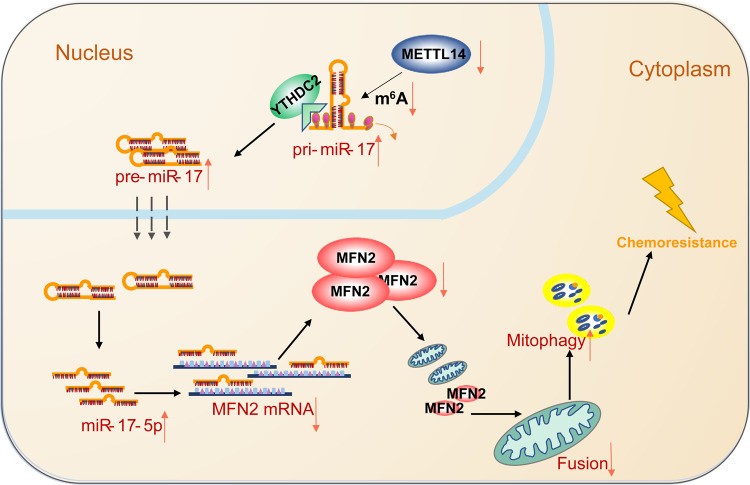
A hypothetical model illustrating the METTL14/miR-17-5p/MFN2 axis induces 5-FU chemoresistance in colorectal cancer. Low expression of METTL14 in CRC resulted in decreased m^6^A modification, which in turn reduced the recognition and binding of pri-miR-17 by YTHDC2, promoted the stability of pri-miR-17 mRNA, and increased the expression of miR-17-5p. Overexpressed miR-17-5p inhibited MFN2, leading to decreased mitochondrial fusion and enhanced mitochondrial fission and mitophagy, which ultimately induced drug resistance.

In summary, we demonstrated that miR-17-5p induced the chemoresistance to 5-FU in CRC. METTL14 was downregulated by a yet-to-be identified mechanism in CRC and decreased the level of m^6^A modification. Low expression of METTL14 in CRC resulted in decreased m^6^A modification level, which in turn reduced the recognition and binding of pri-miR-17 by YTHDC2, promoted the stability of pri-miR-17 mRNA, and increased the expression of miR-17-5p. Furthermore, overexpressed miR-17-5p inhibited MFN2, leading to decreased mitochondrial fusion and enhanced mitochondrial fission and mitophagy, which ultimately induced 5-FU hyposensitivity (Fig. 7). We evaluated the specificity and efficacy of the METTL14/miR-17-5p/MFN2 signaling axis in the regulation of drug sensitivity in CRC. Based on our findings, a new regulatory network involving miR-17-5p might provide novel insights into molecular therapy-based treatment of CRC.

## Supplementary information

Supplementary Information

checklist

## Data Availability

The data that support the findings of this study are available from the corresponding author upon reasonable request.

## References

[1] Sung H, Ferlay J, Siegel RL, Laversanne M, Soerjomataram I, Jemal A (2021). Global Cancer Statistics 2020: GLOBOCAN Estimates of Incidence and Mortality Worldwide for 36 Cancers in 185 Countries. CA Cancer J Clin.

[2] Le DT, Uram JN, Wang H, Bartlett BR, Kemberling H, Eyring AD (2015). PD-1 Blockade in Tumors with Mismatch-Repair Deficiency. N. Engl J Med.

[3] Koopman M, Kortman GAM, Mekenkamp L, Ligtenberg MJL, Hoogerbrugge N, Antonini NF (2009). Deficient mismatch repair system in patients with sporadic advanced colorectal cancer. Br J Cancer.

[4] Walko CM, Lindley C (2005). Capecitabine: a review. Clin Ther.

[5] Vodenkova S, Buchler T, Cervena K, Veskrnova V, Vodicka P, Vymetalkova V (2020). 5-fluorouracil and other fluoropyrimidines in colorectal cancer: Past, present and future. Pharm Ther.

[6] Li K, Chen J, Lou X, Li Y, Qian B, Xu D (2021). HNRNPA2B1 Affects the Prognosis of Esophageal Cancer by Regulating the miR-17-92 Cluster. Front Cell Dev Biol.

[7] Li D, Li C, Chen Y, Teng L, Cao Y, Wang W (2020). LncRNA HOTAIR induces sunitinib resistance in renal cancer by acting as a competing endogenous RNA to regulate autophagy of renal cells. Cancer Cell Int.

[8] Zhang W, Lin J, Wang P, Sun J (2017). miR-17-5p down-regulation contributes to erlotinib resistance in non-small cell lung cancer cells. J Drug Target.

[9] Hasanzadeh M, Movahedi M, Rejali M, Maleki F, Moetamani-Ahmadi M, Seifi S (2019). The potential prognostic and therapeutic application of tissue and circulating microRNAs in cervical cancer. J Cell Physiol.

[10] Zhang Y, Wang S, Lai Q, Fang Y, Wu C, Liu Y (2020). Cancer-associated fibroblasts-derived exosomal miR-17-5p promotes colorectal cancer aggressive phenotype by initiating a RUNX3/MYC/TGF-beta1 positive feedback loop. Cancer Lett.

[11] Meyer KD, Patil DP, Zhou J, Zinoviev A, Skabkin MA, Elemento O (2015). 5’ UTR m(6)A Promotes Cap-Independent Translation. Cell.

[12] Molinie B, Wang J, Lim KS, Hillebrand R, Lu Z-X, Van Wittenberghe N (2016). m(6)A-LAIC-seq reveals the census and complexity of the m(6)A epitranscriptome. Nat Methods.

[13] Fustin J-M, Doi M, Yamaguchi Y, Hida H, Nishimura S, Yoshida M (2013). RNA-methylation-dependent RNA processing controls the speed of the circadian clock. Cell.

[14] Wang X, Lu Z, Gomez A, Hon GC, Yue Y, Han D (2014). N6-methyladenosine-dependent regulation of messenger RNA stability. Nature.

[15] Huang H, Weng H, Chen J (2020). m(6)A Modification in Coding and Non-coding RNAs: Roles and Therapeutic Implications in Cancer. Cancer Cell.

[16] Chen X, Xu M, Xu X, Zeng K, Liu X, Pan B (2020). METTL14-mediated N6-methyladenosine modification of SOX4 mRNA inhibits tumor metastasis in colorectal cancer. Mol Cancer.

[17] Wang H, Wei W, Zhang Z-Y, Liu Y, Shi B, Zhong W (2021). TCF4 and HuR mediated-METTL14 suppresses dissemination of colorectal cancer via N6-methyladenosine-dependent silencing of ARRDC4. Cell Death Dis.

[18] Yang X, Zhang S, He C, Xue P, Zhang L, He Z (2020). METTL14 suppresses proliferation and metastasis of colorectal cancer by down-regulating oncogenic long non-coding RNA XIST. Mol Cancer.

[19] Sun Y, Li S, Yu W, Zhao Z, Gao J, Chen C, et al. N(6)-methyladenosine-dependent pri-miR-17-92 maturation suppresses PTEN/TMEM127 and promotes sensitivity to everolimus in gastric cancer. Cell Death Dis. 2020;11:836. 10.1038/s41419-020-03049-w PMC7547657 33037176

[20] Ma L, Chen T, Zhang X, Miao Y, Tian X, Yu K (2021). The m(6)A reader YTHDC2 inhibits lung adenocarcinoma tumorigenesis by suppressing SLC7A11-dependent antioxidant function. Redox Biol.

[21] Zhou B, Liu C, Xu L, Yuan Y, Zhao J, Zhao W, et al. N(6) -Methyladenosine Reader Protein YT521-B Homology Domain-Containing 2 Suppresses Liver Steatosis by Regulation of mRNA Stability of Lipogenic Genes. Hepatology. 2021;73:91–103. 10.1002/hep.31220 32150756

[22] Mao Y, Dong L, Liu X-M, Guo J, Ma H, Shen B (2019). m(6)A in mRNA coding regions promotes translation via the RNA helicase-containing YTHDC2. Nat Commun.

[23] Qi M, Dai D, Liu J, Li Z, Liang P, Wang Y (2020). AIM2 promotes the development of non-small cell lung cancer by modulating mitochondrial dynamics. Oncogene.

[24] Lin XH, Qiu BQ, Ma M, Zhang R, Hsu SJ, Liu HH (2020). Suppressing DRP1-mediated mitochondrial fission and mitophagy increases mitochondrial apoptosis of hepatocellular carcinoma cells in the setting of hypoxia. Oncogenesis.

[25] Lang L, Loveless R, Dou J, Lam T, Chen A, Wang F (2022). ATAD3A mediates activation of RAS-independent mitochondrial ERK1/2 signaling, favoring head and neck cancer development. J Exp Clin Cancer Res.

[26] Zhou Y, Wang Q, Deng H, Xu B, Zhou Y, Liu J (2022). N6-methyladenosine demethylase FTO promotes growth and metastasis of gastric cancer via mA modification of caveolin-1 and metabolic regulation of mitochondrial dynamics. Cell Death Dis.

[27] Hinshaw DC, Hanna A, Lama-Sherpa T, Metge B, Kammerud SC, Benavides GA (2021). Hedgehog Signaling Regulates Metabolism and Polarization of Mammary Tumor-Associated Macrophages. Cancer Res.

[28] Padder RA, Bhat ZI, Ahmad Z, Singh N, Husain M (2020). DRP1 Promotes BRAF-Driven Tumor Progression and Metabolic Reprogramming in Colorectal Cancer. Front Oncol.

[29] Chen M, Ye K, Zhang B, Xin Q, Li P, Kong A-N (2019). Paris Saponin II inhibits colorectal carcinogenesis by regulating mitochondrial fission and NF-κB pathway. Pharm Res.

[30] Yin K, Lee J, Liu Z, Kim H, Martin DR, Wu D (2021). Mitophagy protein PINK1 suppresses colon tumor growth by metabolic reprogramming via p53 activation and reducing acetyl-CoA production. Cell Death Differ.

[31] Kraus F, Roy K, Pucadyil TJ, Ryan MT (2021). Function and regulation of the divisome for mitochondrial fission. Nature.

[32] Drake LE, Springer MZ, Poole LP, Kim CJ, Macleod KF (2017). Expanding perspectives on the significance of mitophagy in cancer. Semin Cancer Biol.

[33] Ren TJ, Liu C, Hou JF, Shan FX (2020). CircDDX17 reduces 5-fluorouracil resistance and hinders tumorigenesis in colorectal cancer by regulating miR-31-5p/KANK1 axis. Eur Rev Med Pharm Sci.

[34] Wang H, Wang X, Zhang H, Deng T, Liu R, Liu Y (2021). The HSF1/miR-135b-5p axis induces protective autophagy to promote oxaliplatin resistance through the MUL1/ULK1 pathway in colorectal cancer. Oncogene.

[35] Zheng G-L, Liu Y-L, Yan Z-X, Xie X-Y, Xiang Z, Yin L (2021). Elevated LOXL2 expression by LINC01347/miR-328-5p axis contributes to 5-FU chemotherapy resistance of colorectal cancer. Am J Cancer Res.

[36] Mastropasqua F, Marzano F, Valletti A, Aiello I, Di Tullio G, Morgano A (2017). TRIM8 restores p53 tumour suppressor function by blunting N-MYC activity in chemo-resistant tumours. Mol Cancer.

[37] Oun R, Moussa YE, Wheate NJ (2018). The side effects of platinum-based chemotherapy drugs: a review for chemists. Dalton Trans.

[38] Yamashita K, Miyata H, Makino T, Masuike Y, Furukawa H, Tanaka K (2017). High Expression of the Mitophagy-Related Protein Pink1 is Associated with a Poor Response to Chemotherapy and a Poor Prognosis for Patients Treated with Neoadjuvant Chemotherapy for Esophageal Squamous Cell Carcinoma. Ann Surg Oncol.

[39] Kim SJ, Syed GH, Khan M, Chiu WW, Sohail MA, Gish RG (2014). Hepatitis C virus triggers mitochondrial fission and attenuates apoptosis to promote viral persistence. Proc Natl Acad Sci USA.

[40] Luo M, Huang Z, Yang X, Chen Y, Jiang J, Zhang L (2022). PHLDB2 Mediates Cetuximab Resistance via Interacting With EGFR in Latent Metastasis of Colorectal Cancer. Cell Mol Gastroenterol Hepatol.

[41] Peng W, Li J, Chen R, Gu Q, Yang P, Qian W (2019). Upregulated METTL3 promotes metastasis of colorectal Cancer via miR-1246/SPRED2/MAPK signaling pathway. J Exp Clin Cancer Res.

[42] Zhang J, Bai R, Li M, Ye H, Wu C, Wang C (2019). Excessive miR-25-3p maturation via N(6)-methyladenosine stimulated by cigarette smoke promotes pancreatic cancer progression. Nat Commun.

[43] Ni W, Yao S, Zhou Y, Liu Y, Huang P, Zhou A, et al. Long noncoding RNA GAS5 inhibits progression of colorectal cancer by interacting with and triggering YAP phosphorylation and degradation and is negatively regulated by the m(6)A reader YTHDF3. Mol Cancer. 2019;18:143. 10.1186/s12943-019-1079-y PMC6794841 31619268

[44] Wang S, Gao S, Zeng Y, Zhu L, Mo Y, Wong CC (2022). N6-Methyladenosine Reader YTHDF1 Promotes ARHGEF2 Translation and RhoA Signaling in Colorectal Cancer. Gastroenterology.

[45] Wang X, Zhao BS, Roundtree IA, Lu Z, Han D, Ma H (2015). N(6)-methyladenosine Modulates Messenger RNA Translation Efficiency. Cell.

[46] Xu Y, He X, Wang S, Sun B, Jia R, Chai P (2022). The m(6)A reading protein YTHDF3 potentiates tumorigenicity of cancer stem-like cells in ocular melanoma through facilitating CTNNB1 translation. Oncogene.

[47] Lv W, Tan Y, Xiong M, Zhao C, Wang Y, Wu M (2021). Analysis and validation of m6A regulatory network: a novel circBACH2/has-miR-944/HNRNPC axis in breast cancer progression. J Transl Med.

