# Supplementary material for: METTL14-dependent maturation of pri-miR-17 regulates mitochondrial homeostasis and induces chemoresistance in colorectal cancer
Source: Cell Death Dis. 2023 Feb 21;14(2):148. doi: 10.1038/s41419-023-05670-x (PMC9944299; doi:10.1038/s41419-023-05670-x)
Supplement: Supplementary file 1 — Supplementary Information [file 41419_2023_5670_MOESM1_ESM.docx]

**Supplementary Information**

**METTL14-dependent maturation of pri-miR-17 regulates mitochondrial homeostasis and induces chemoresistance in colorectal cancer**

**Sun et al.**


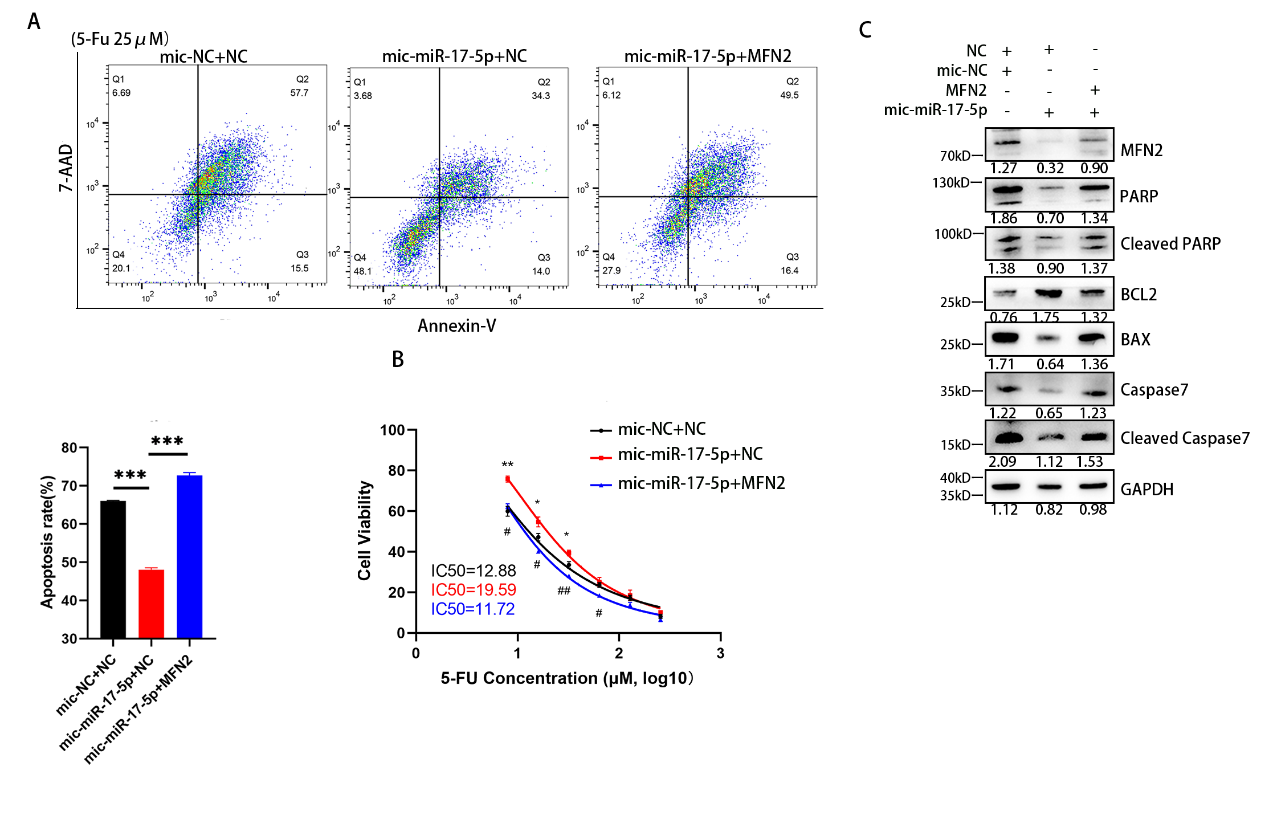


**Supplementary Figure 1 Co-expression of MFN2 promotes apoptosis in CRC cells.**

(A, B) Rescue experiments on MFN2 and miR-17-5p: IC50 and Annexin V-PE/7AAD apoptosis of 5-FU in HCT116 cells. (C) Western blot analysis of MFN2, PARP, cleaved PARP, BAX, Caspase7 and cleaved Caspase7 of rescue experiments. The results shown were representative of at least 3 independent experiments. Statistical significance in (A-B) was determined by one-way ANOVA with Dunnett’s multiple comparisons test. The grey value of protein bands has been quantified by Image J.


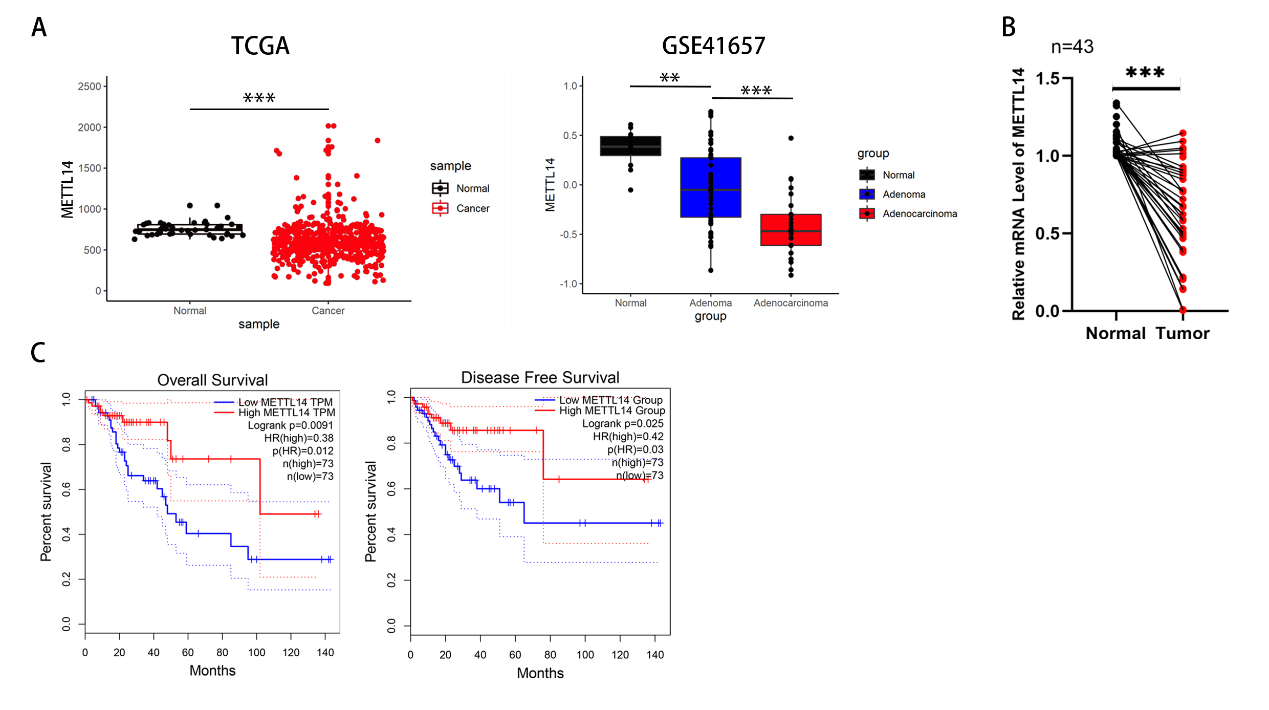


**Supplementary Figure 2 METTL14 is low-expression in CRC tissues.**

(A) Expression of METTL14 in the TCGA CRC cohort as well as the GSE41657 adenoma and adenocarcinoma cohorts. adj.P (TCGA) ˂ 0.0001. adj.P (GSEA. normal vs. adenoma) = 0.0019; adj.p (GSEA. adenoma vs. adenocarcinoma) = 0.0005. (B) Relative expression of METTL14 in 43 pairs of CRC tissues from a clinical cohort. P ˂ 0.0001. Statistical significance was assessed by student’s t-test. (C) Analysis of overall survival and disease-free survival of METTL14 from GEPIA databases.


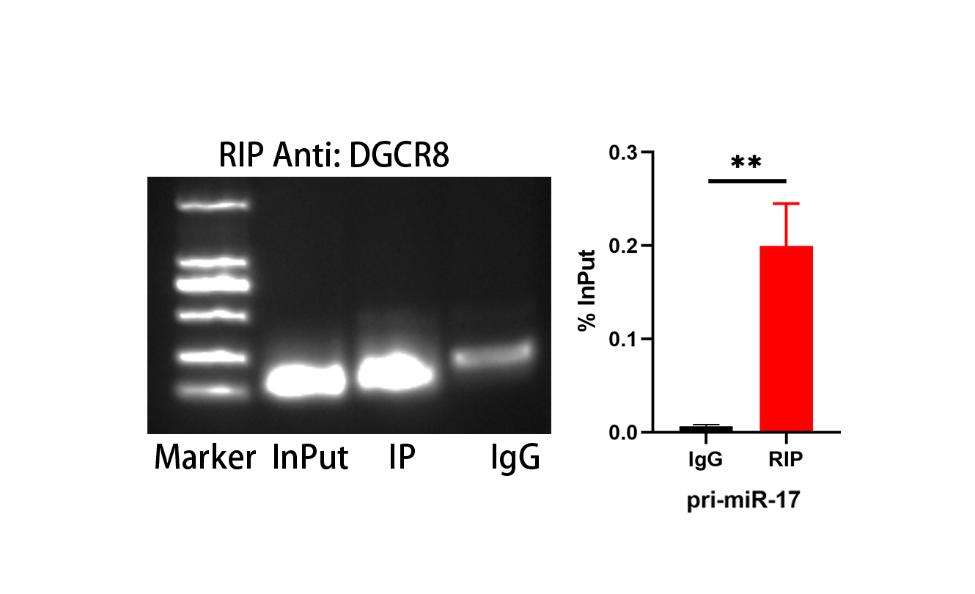


**Supplementary Figure 3 RIP shows DGCR8 directly binds pri-miR-17.**

RIP and RIP-qPCR assay using DGCR8-specifc antibody and IgG control antibody to measure the enrichment of DGCR8 binding to pri-miR-17. P = 0.0018. Results shown were representative of at least 3 independent experiments. Statistical significance was assessed by student’s t-test.


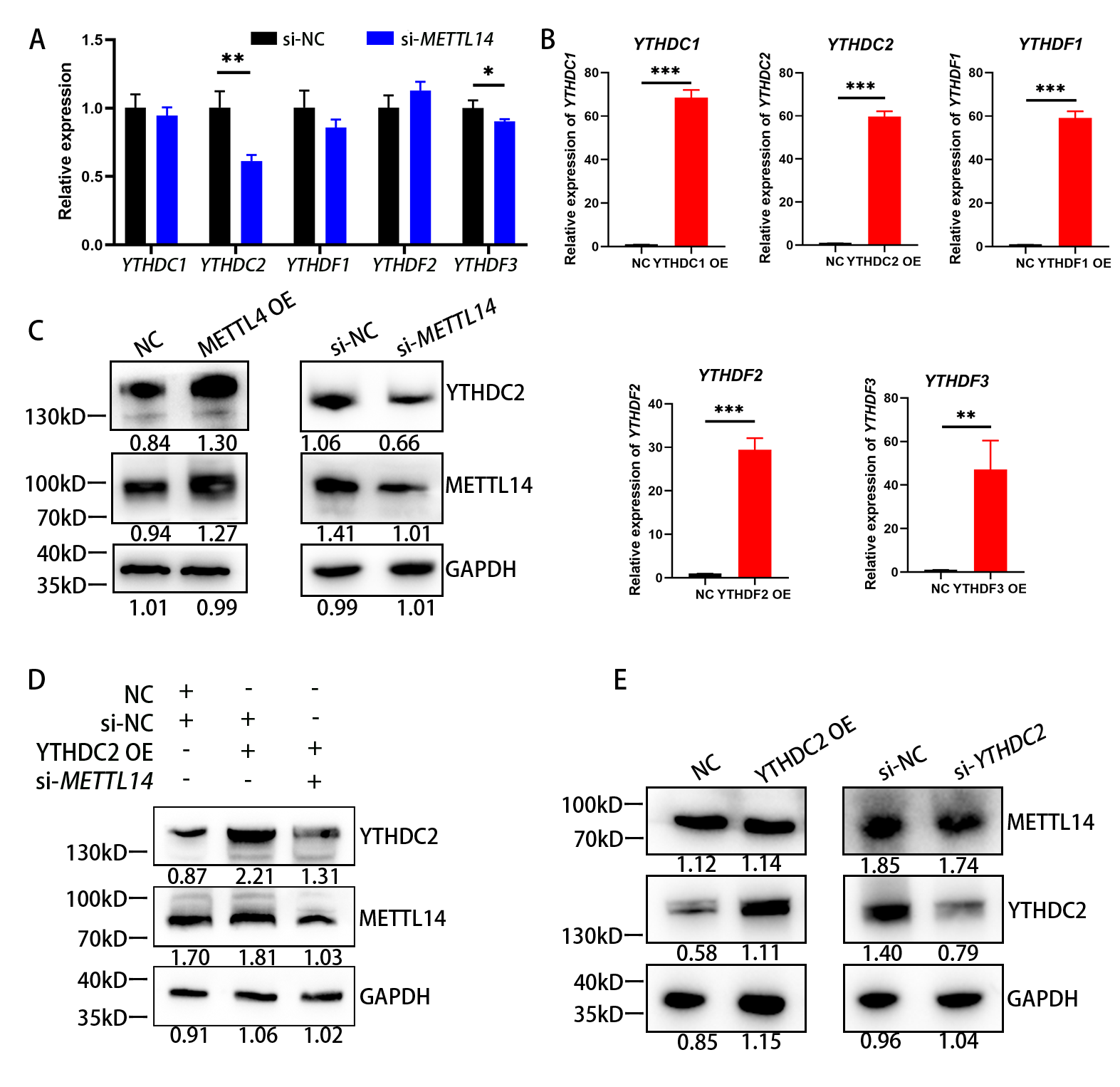


**Supplementary Figure 4 The expression of YTHDC2 is positively correlated with METTL14 in CRC cells.**

(A) RT-PCR analysis of m^6^A readers in HCT116 cells after METTL14 knockdown. P (*YTHDC2*) = 0.0059; P (*YTHDF3*) = 0.0428. (B) Overexpression efficiency of YTHDC1-2, YTHDF1-3 plasmids. P (NC vs. YTHDC1-2 OE, YTHDF1-2 OE) all < 0.0001; P (NC vs. YTHDF3 OE) = 0.0039. (C) Western blot analysis of YTHDC2 after METTL14 knockdown or overexpression. (D) Western blot analysis of YTHDC2 after HCT116 cells transfected of YTHDC2 plasmid and cotransfected of si-*METTL14* and YTHDC2 plasmid. (E) Western blot analysis of METTL14 after YTHDC2 knockdown or overexpression. Results shown were representative of at least 3 independent experiments. Statistical significance in (A), (B) was assessed by student’s t-test. *, P ˂ 0.05; **, P ˂ 0.01; ***, P ˂ 0.001. Error bars, SD. The grey value of protein bands has been quantified by Image J.


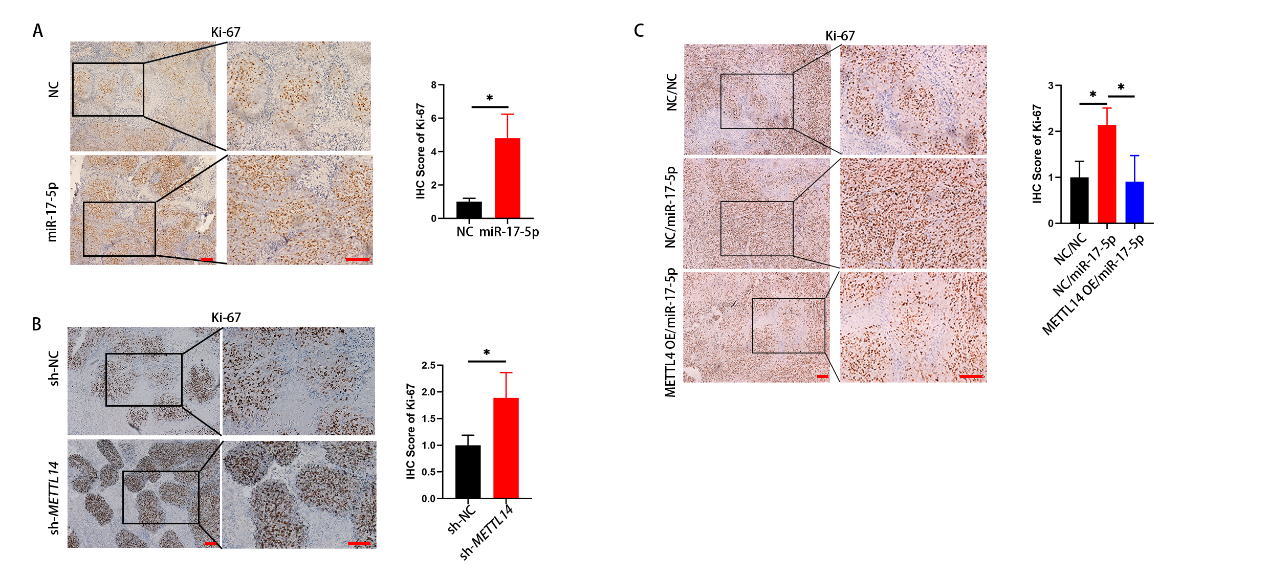


**Supplementary Figure 5 Immunohistochemistry analysis of Ki-67 in xenograft experiments.**

Immunohistochemistry analysis of Ki-67 in (A) miR-17-5p-overexpressed and control groups, P = 0.0103; (B) sh-*METTL14* and sh-NC groups, P = 0.0398; as well as (C) miR-17-5p-overexpressed, and co-expression of METTL14 OE/miR-17-5p and control group, P (NC/NC vs. NC/miR-17-5p) = 0.0335; P (NC/miR-17-5p vs. miR-17-5p/METTL14 OE) = 0.0241. Results shown were representative of at least 3 independent experiments. Statistical significance in (A-B) was assessed by student’s t-test. Statistical significance in (C) was determined by one-way ANOVA with Dunnett’s multiple comparisons test. The IHC score has been quantified by Image J.


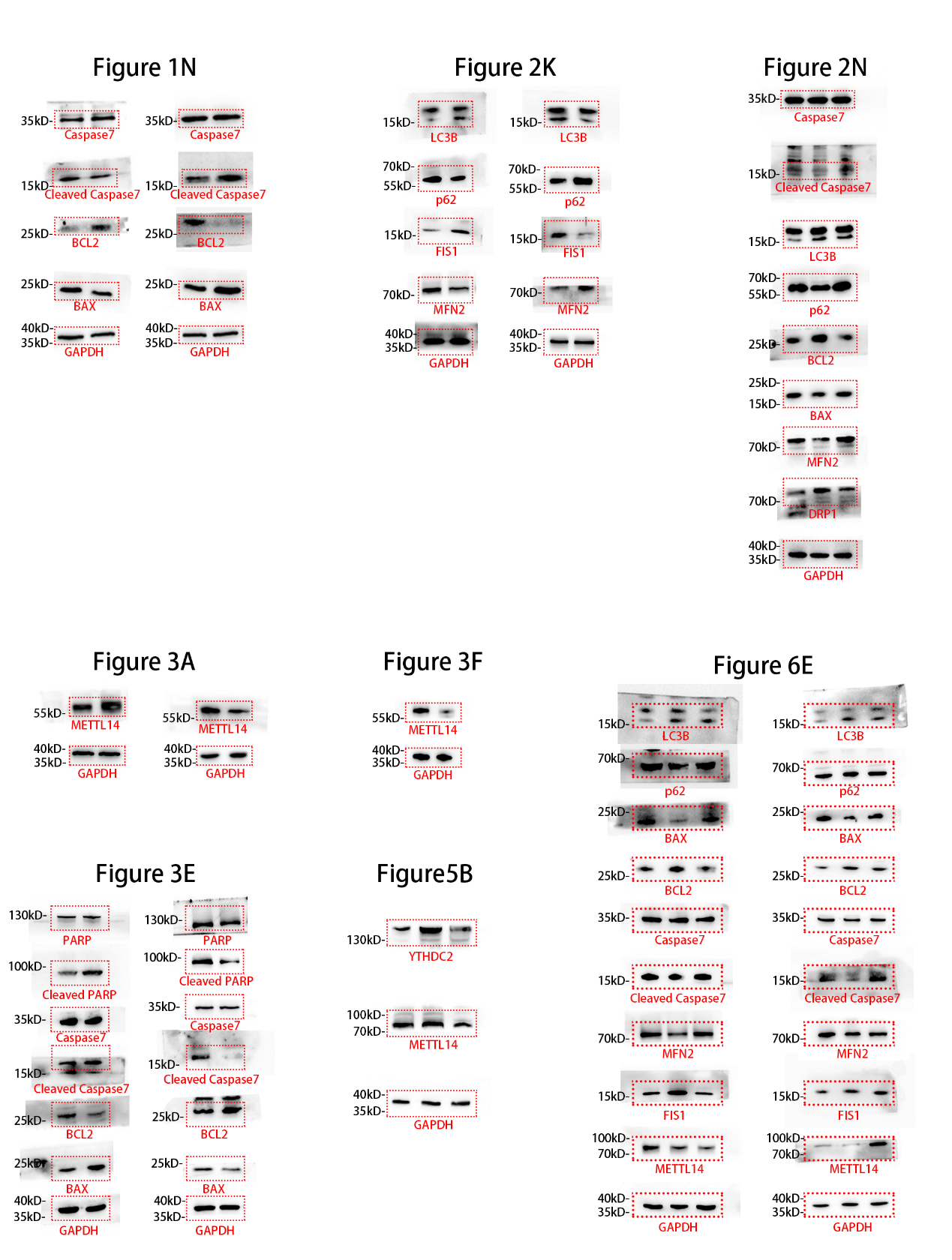


**Supplementary Figure 6 The uncropped scans of western blots from the main figures**

**Supplementary Materials and Methods**

**RNA isolation, reverse transcription, and quantitative real-time PCR (RT–PCR)**

Total RNA was extracted using TRIzol reagent (Takara, Japan). To quantify gene expression, the isolated total RNA was polyadenylated and subjected to reverse transcription using PrimeScript RT Master Mix (Takara, Japan). RT–PCR was performed in triplicate using TB Green Premix Ex Taq II (Takara, Japan). miRNA levels were detected using a PrimeScript RT Reagent Kit with gDNA Eraser (Perfect Real Time; Takara) and a Roche LightCycler 480 Real-Time PCR System (Roche Diagnostics, Germany). Relative gene expression levels were calculated by using the 2-ΔΔCt method. The ΔCt value of each sample was calculated using GAPDH or U6 as an endogenous control gene. The primers are shown in Supplementary Table S2.

**Cell apoptosis assay**

Cells were seeded in six-well plates at a concentration of 2 × 10^5^ cells per well overnight and treated with 5-FU (25 μM; Selleck, USA) for 48 h. Then, the cells were harvested, washed, and stained with Annexin V-PE/7-AAD reagents according to the manufacturer’s protocol (KeyGen, China). Apoptosis was measured by flow cytometry (BD, USA).

**Cell growth**

Cells were seeded in 96-well plates at a concentration of 5000 cells per well overnight and treated with different concentrations of 5-FU for 48 h. The culture medium in each well was replaced with a mixture containing 10 μl CCK8 (Meilunbio, China) and 90 μl culture medium. Two hours later, the absorbance was measured at an OD value of 450 nm using an enzyme microplate reader (Molecular Devices, USA). The half-maximal inhibitory concentration (IC50) was calculated with GraphPad Prism 8 (GraphPad Software, USA).

**EdU proliferation assay**

Cells were seeded in 96-well plates at 8000 cells per well, incubated overnight and treated with 5-FU (25 μM) for 48 h. EdU staining was performed according to the manufacturers’ instructions (Ribobio, China).

**Animals and a tumor growth assay**

Female BALB/c nude mice (3 weeks old) were purchased from the Guangdong Medical Laboratory Animal Center and housed under 12-h light/dark cycles with ad libitum access to water and food. All animal handling and experimental procedures were approved by the Animal Experimental Ethics Committee of Southern Medical University. In each animal experiment, mice were randomly allocated to different groups. To establish a xenograft model, 5×10^6^ SW480 cells diluted in 100 μl PBS were subcutaneously inoculated into the back of mice, and tumor growth was measured every 3 days. Beginning at 72 h after inoculation, the mice were injected intraperitoneally with 5-FU (30 mg/kg) twice weekly for 21 days (6 doses in total). The mice were sacrificed after treatment, and the tumors were dissected, weighed, and fixed with 4% paraformaldehyde for further study. Tumor volume was calculated using the following formula: V = 0.52 × D × d^2^, where V represents the volume, D represents the vertical diameter, and d represents the horizontal diameter.

**Analysis of public databases**Raw gene expression data for CRC were downloaded from The Cancer Genome Atlas (TCGA) (http://cancergenome.nih.gov) and Gene Expression Omnibus (GEO) database. The independent data sets GSE41657 and GSE81005 were analyzed in this study.

**Determination of the mitochondrial DNA (mtDNA) copy number**

Total genomic DNA was extracted using the Steady Pure Universal Genomic DNA Extraction Kit (Accurate Biotechnology, China). Then, 100 ng DNA and both the forward and reverse primers for mitochondrially encoded cytochrome c oxidase-2 (MT-CO2) (10 µM per primer) were added to the reaction together with TB Green Premix Ex Taq II (Takara). Relative gene expression levels were calculated by using the 2-ΔΔCt method. The ΔCt value of each sample was calculated using GAPDH as an endogenous control gene. The primers are shown in Supplementary Table S2.

**Mitochondrial membrane potential and an immunofluorescence assay**

To measure the mitochondrial membrane potential, cells were incubated with the fluorescent mitochondrial probe JC-1 according to the manufacturer’s instructions (Beyotime, China). To label the mitochondria and lysosomes, cells were cultured with MitoTracker Red or LysoTracker Green (Beyotime).

**Mitochondrial morphology**

After treatment with the mitophagy inhibitor Mdivi-1 (5 μg/ml, Selleck) for 48h, cells were cultured with MitoTracker Red (Beyotime) and observed by confocal laser scanning microscopy (LSM980; Zeiss, Germany).

After transfection for 48h, cells were harvested, pelleted, and fixed in 2.5% glutaraldehyde. Ultrathin sections were stained with uranyl acetate and lead citrate and observed using a transmission electron microscope (FEI, USA).

**Supplementary Table 1** **Sequences of miRNA mimics, inhibitor and si-RNA**

|  | sense (5’-3’) | antisense (5’-3’) |
| --- | --- | --- |
| has-miR-17-5p mimics | CAAAGUGCUUACAGUGCAGGUAG | ACCUGCACUGUAAGCACUUUGUU |
| mic-NC | UUCUCCGAACGUGUCACGUTT | ACGUGACACGUUCGGAGAATT |
| has-miR-17-5p inhibitor | CUACCUGCACUGUAAGCACUUUG | / |
| i-NC | CAGUACUUUUGUGUAGUACAA | / |
| si-*METTL14* | CAGCAUUGGUGCCGUGUUATT | UAACACGGCACCAAUGCUGTT |
| si-*YTHDC2* | GCUUAAGACAAUAGAUGCATT | UGCAUCUAUUGUCUUAAGCTT |
| si-NC | UUCUCCGAACGUGUCACGTT | ACGUGACACGUUCGGAGAATT |

**Supplementary Table 2 Primers for RT-PCR**

|  | Forward Primer | Reverse Primer |
| --- | --- | --- |
| GAPDH | GCACCGTCAAGGCTGAGAAC | TGGTGAAGACGCCAGTGGA |
| METTL14 | GTTGGAACATGGATAGCCGC | CAATGCTGTCGGCACTTTCA |
| pri-miR-17 | TGCTTACAGTGCAGGTAGTGATA | CAAAAAGCACTCAACATCAGCAGG |
| pre-miR-17 | GCAGGAAAAAAGAGAACATCACC | TGGCTTCCCGAGGCAG |
| miR-17-5p | GCGCAAAGTGCTTACAGTGC | AGTGCAGGGTCCGAGGTATT |
| U6 | AGAGAAGATTAGCATGGCCCCTGC | ATCCAGTGCAGGGTCCGAGG |
| YTHDF1 | TCCGATTCCATACCTCACCACCTAC | AACCTGTGCTGATAGATGTTGTTCCC |
| YTHDC1 | GCAAGCAGATCCAGCCAGTCTTC | TCTTCCACTCCTTCCTCCTCATTCTC |
| YTHDF2 | TAACAAGAGACTGGATGCTGCTTATCG | GATTTCATTTCTGCCACGCCACAG |
| YTHDF3 | AGCAGCAGTGGTATGACTAGCATTG | GGGTTTAAGTTTCGGTTGAGGTTTGG |
| YTHDC2 | GAGGCCTTTCTGGTGACCTC | TTGTTGAGTCGCCCACTTGT |
| MT-ND1 | AACATACCCATGGCCAACCT | AGCGAAGGGTTGTAGTAGCCC |
| MT-CO2 | CAAACCTACGCCAAAATCCA | GAAATGAATGAGCCTACAGA |
